# José Roberto Ferreira: an intermediary between international health and global health, 1959-2019

**DOI:** 10.1590/S0104-59702025000100017en

**Published:** 2025-05-02

**Authors:** Erica Kastrup, Analice Pinto Braga, Gabriel Lopes

**Affiliations:** i International Cooperation Analyst, Centro de Relações Internacionais em Saúde/Fiocruz. Rio de Janeiro – RJ – Brazil. erica.kastrup@fiocruz.br; ii International Relations Advisor, Fiocruz. Rio de Janeiro – RJ – Brazil. analicepbraga@gmail.com; iii Post-Doctoral Research Intern, Casa de Oswaldo Cruz/Fiocruz. Rio de Janeiro – RJ – Brazil. lopes-gabriel@hotmail.com

**Keywords:** Global health, International health, South-South cooperation, Health education, José Roberto Ferreira (1934-2019

## Abstract

This article presents José Roberto Ferreira’s contribution to national and international discussions on the training of health professionals and his contributions to the construction of a critical proposal for South-South cooperation in health. The article argues that Ferreira advocated that health education, developed based on the particular realities of less developed countries, would be a path to emancipation. Initially, we will present Ferreira’s training and early works in the field of education (1959-1969); his international experiences and contacts with important figures in Latin American social medicine (1969-1996); and, finally, his work at Fiocruz within the framework of foreign policy for the development of structuring cooperation in health (1996-2019).

Health has gained an important place in Brazilian foreign policy since the start of the twenty-first century, and in the specific case of Luiz Inácio Lula da Silva’s government, actions in this field constitute one of the main instruments of a foreign policy intended to bring Brazil closer to other developing countries in the Global South. The idea was to shape the country as an emerging power capable of supporting the development of other nations through technical cooperation and exchange of experiences on successful national public policies, especially the Unified Health System (Sistema Único de Saúde, SUS) (Almeida et al., 2023; Buss, 2018; Cueto, Lopes, 2023). Within this context, a group of Brazilian health experts published an article in which they introduced the concept of structuring South-South cooperation in health (SSSC), along with topics and relationship models to guide Brazil’s international health activities (Almeida et al., 2010).

Innate to the notion of SSSC is the criticism that international cooperation in health historically lacked efficacy because it was formulated outside of the areas where it was applied, creating dissonance between the activities and the real needs of the countries they were intended to benefit. With this in mind, SSSC proposed guiding Brazilian cooperation by presented demands, establishing partnerships and respecting the specific rationales of the partners. To do so, beneficiaries must be actively involved in designing the projects, avoiding passive transfer of knowledge and promoting empowerment of local public authorities with regard to interventions carried out within their territories. The central themes of this cooperation in health were training human resources and institutional strengthening so the results of these programs and projects structure institutions which in turn could serve as the pillars of local health systems and then be capable of autonomous development processes through health (Almeida et al., 2010; Ferreira et al., 2016).

This model of cooperation dialogued with the agenda of restructuring health systems which emerged in the first decade of the 2000s, associated with the notion of effective development aid. The article by Almeida et al. (2010) points out that the ideas about the concept of structuring cooperation came from the process of developing projects Brazil was offering to other countries in the Global South as part of the assertive and ambitious foreign policy adopted during the Lula da Silva administration (2003-2010). In fact, this was an auspicious time for the government to employ the proposal of structuring cooperation and disseminate its ideology. This concept of international cooperation was conceived by the Brazilian physician José Roberto Ferreira, based on his personal experiences and a web of relationships that permeated his career that spanned international and global health.

Along these lines, here we argue that Ferreira played an essential role in laying the foundations for what has become known as “structuring South-South cooperation;” this concept was systematized and developed as part of a health-related Brazilian foreign policy agenda and published in March 2010 in the *Revista Eletrônica de Comunicação, Informação & Inovação em Saúde – Reciis,* a publication of the Institute for Communication and Scientific and Technological Information in Health at the Oswaldo Cruz Foundation (Fiocruz), in an article which listed Ferreira as one of the authors (Almeida et al., 2010).^1^

To outline our argument, we will focus on three key periods in Ferreira’s career. The first, from 1959 to 1969, depicts the beginning of the young doctor’s work, which combined research in the field of cardiac surgery with the development of medical school projects to bring teaching closer to health services. The second refers to his work at the Pan American Health Organization (PAHO) from 1969 to 1996, when he worked with important figures in the Latin American social medicine movement and, from an international perspective, began to argue that health education developed according to the specific realities of less-developed countries would serve to emancipate these countries from a system of international cooperation that reproduced asymmetries. In this sense, he constructed a southern perspective that associated the practice of international cooperation in health with ideas of emancipation. The third period, from 1996 to 2019, covers the years he worked at the Oswaldo Cruz Foundation, where he was able to put these ideas into practice through South-South cooperation (SSC) projects developed by the Brazilian government.

José Roberto Ferreira died on December 25, 2019; the sources used here to reconstruct his career include his own life story, a manuscript provided by his daughter in 2020. Analysis of this material was supplemented with interviews he gave to the History and Health Observatory at the Casa de Oswaldo Cruz and articles, published books and other academic works that discuss health cooperation offered by Brazil, which will be mentioned throughout the text.

## Early career and interest in institutional development

José Roberto Ferreira began his career as a doctor in the late 1950s, when he worked in cardiac surgery at the hospital of Ipanema. His father was a renowned tuberculosis specialist and full member of Brazil’s National Academy of Medicine (Academia Nacional de Medicina), which facilitated Ferreira’s connections with the elite of Rio de Janeiro’s medical society in the 1950s and 1960s. Ferreira was interested in the development of medical technologies and academic research,^2^ and through his father’s contacts became editor of the *Jornal Brasileiro de Medicina* (Brazilian Journal of Medicine) in 1959, two years after graduating from the University of Brazil in Rio de Janeiro. Interested in modernizing surgery, he traveled to the United States as an assistant to Lucio Galvão, a surgeon emeritus of the Academy of Medicine, to research a cardiopulmonary bypass technique he would bring back to Brazil.

His initiation into the field of medical education management and administration arose through Galvão, who invited him to serve as his replacement on a working group created to define the basic guidelines for the reform of the University of Brazil. Ferreira himself stated that unlike his young assistant, Galvão had no interest in administrative activities and for this reason asked to be replaced. Within this group, Ferreira created a web of relationships that would drive his interest in the development of medical teaching institutions.

One member of the group was Ernani Braga, who was working on a Rockefeller Foundation project with the Coordination for the Improvement of Higher Education Personnel (Coordenação de Aperfeiçoamento de Pessoal de Nível Superior, CAPES) in Brazil which involved promoting the training of physicians and nurses in the country and collaborating with other Latin American countries where the foundation was carrying out the same project. In 1962 the Pan-American Federation of Medical Schools was created, and Braga was invited to be the executive director of this new organization. An office was opened in Rio de Janeiro, in a space provided at PAHO’s Brazilian headquarters, and the new director invited Ferreira to be his assistant. One responsibility of the federation (under the patronage of PAHO, the Rockefeller Foundation and other American philanthropic foundations) was to encourage the creation of national networks of medical schools, which was consolidated in Brazil with the foundation of the Brazilian Association of Medical Education the following year, with Ferreira appointed as its executive director. At that time, PAHO also founded an area dedicated to training human resources for health in Brazil.

Another colleague in the working group for reforms at the University of Brazil was Durmeval Trigueiro Mendes, at that time the director of higher education at the Ministry of Education and Culture (MEC), who invited Ferreira to serve as secretary of the Planning Commission for Medical Training (Comissão de Planejamento da Formação de Médicos, CPFM), a group that was being set up for this task within the MEC. Ferreira accepted the position, which consisted of analyzing requests to open new medical schools, acting as an advisor to a movement to expand medical education which was taking place in Brazil throughout the 1960s.

Between 1965 and 1970, 33 new medical schools were authorized to open with government subsidies (Haddad et al., 2010). At the same time, on the regional level PAHO had been discussing preventive medicine since the mid-1950s,^3^ with a proposal to include a subject within the medical school curriculum that would combine teaching biology with social science. This movement can be understood as a process of resisting the advance of the biomedical model in health care; throughout the 1960s, it gradually spread across Brazil with the creation of preventive medicine centers and departments within university instruction (Arouca, 2003; Escorel, 1999).

Within the CPFM, Ferreira made recommendations and adjustments to the proposals, and drafted projects for structuring and operating medical schools. This work, which took place at the same time as his activities at the Brazilian Association of Medical Education, catalyzed exchanges between Brazilian medical schools. One of the schools being created was the National School of Public Health (Escola Nacional de Saúde Pública, ENSP) at the Oswaldo Cruz Foundation; Ferreira drew up its bylaws in 1965.

The following year, Ferreira was invited to take part in creating the new School of Medicine at the University of Brasilia (UnB), and was among those responsible for constructing a project considered innovative for also incorporating the teaching of nursing and dentistry into the common basic subjects, guiding the curricula along integrated lines of biomedical teaching, public health and clinical practice, which established a new course profile based on integrating teaching with community medical services.

The project was based on the idea of integrating teaching and care (known in Portuguese as *integração docente-assistencial,* IDA), an approach to health education that emphasized the need for greater integration between training and practice in health services (Pires-Alves et al., 2010; Pires-Alves, Paiva, Hochman, 2008). In general terms, this approach promoted teaching based on multiprofessional integration with an emphasis on primary care services, and promoted interdisciplinarity in medical education. The IDA model and preventive medicine used different perspectives to argue that it was important to broaden the physician’s outlook towards more holistic approaches to the health/disease process.

Later that year, Ferreira was invited by the dean of UnB at that time to serve as vice-dean; enthusiastic about the university project created by Darcy Ribeiro with the backing of the Minister of Education (whom Ferreira had met on the former MEC commission), he accepted the invitation and moved to the new federal capital. At the time, Ernani Braga had moved to Geneva to become director of the Human Resources Development Department at the World Health Organization (WHO), which at that time was headed by his longtime friend, the Brazilian Marcolino Candau. At WHO, Braga began to promote UnB’s School of Health Sciences as a model, which led José Roberto Ferreira to be known as an expert in shaping schools of medicine and public health as well as for having created a movement for international authorities to visit the university campus. In 1967, Braga invited Ferreira and a Canadian consultant to advise on establishing a medical school in the Republic of Cameroon, a former French and English colony that had gained independence a few years earlier, an experience that was Ferreira’s initiation into the field of international cooperation.

On his first assignment in Africa, as a WHO consultant, Ferreira initially thought it would not be possible to create a university program in such precarious conditions, but he was dissuaded by Marcolino Candau himself, who pointed out that it was necessary to find a model capable of meeting this challenge.

Ferreira drew up the proposal to create the program, which was approved by WHO and financed by the World Bank in 1968, a time when demonstrations against Brazil’s military dictatorship were intensifying, especially against Institutional Act No. 5 (AI5) which was decreed by General Artur da Costa e Silva in December of that year. The sieges against university students and the university itself led Ferreira to resign as vice-dean of UnB.

In his own words, the repercussions of his work in Cameroon at WHO headquarters in Geneva was what prompted PAHO director Abraham Horwitz to invite him to serve as a regional consultant for medical education at the organization’s headquarters in Washington.

## International health, South-South cooperation and the idea of a “native” focus

Ferreira arrived at PAHO headquarters in Washington D.C. in January 1969. From that time on, his professional practice began to combine concern with health cooperation models with the progressive principles of public health, which acquired characteristics specific to the Americas and formed what became known as Latin American social medicine (Nunes, 2013; Tajer, 2003). Since its creation, PAHO had been dedicated to discussing the working profiles and careers of doctors and other health professionals in the region, and at that time a center of thinking that sought to associate knowledge in the social sciences with the training of health professionals was forming in the Human Resources Division at the organization’s headquarters in the US capital. In the following years, this center held debates and developed ideas, theses, teaching programs and cooperation projects that focused on the social vision of health and became a point of tension against the hegemony of the biomedical model in international health.

The ideas of Latin American social medicine contrasted with proposals to combat and even eradicate diseases without changing the social conditions in which people lived. In this sense, they were imbued with an ideal of social transformation that translated into defense of equitable access to health services, provision of services as a responsibility of the state, and the importance of social sciences and politics in public health (Nunes, 2013, 2015; Tajer, 2003). The Latin American social medicine movement was not restricted to the area of human resources, but its origins, main formulations and sustainability were found there, in teaching and research programs that defined public health as anchored in social, economic and political determination of the health/disease process (Castro, 2010).

These ideas about the organization of services, health education, and how to promote cooperation between countries flourished in Latin America in the 1970s under the influence of social and political movements taking place in other sectors. The 1970s were a fruitful decade for the cooperation movement between the “third world” countries, which began a process of rapprochement in the 1950s when processes of independence began in Asia and Africa and further developed in the 1960s through a rationale of distancing themselves from East-West ideological conflict and contesting the domination of the North over the South as well as colonialism.

At that time, this movement grew as the decolonization process intensified, anchored in the idea of solidarity between nations on the periphery and the fight against injustice that structured the international system of countries, and came to be known as the Non-Aligned Movement (NAM). These countries wanted to maintain sovereignty and self-determination and organize alliances in order to reduce dependence on industrialized states, seeking shared solutions based on their own characteristics, which gave rise to a Third Worldist agenda that would develop in the following years alongside the Cold War (Almeida, 2017; Pereira, Medeiros, 2015; Pino, 2014).

This movement was based on criticism of the North-South cooperation models, which were seen as unidirectional, assistance-based, ineffective and interest-driven, and affirmed Technical Cooperation between Developing Countries as an instrument for autonomy of less-developed countries and a complementary to North-South cooperation. The central idea was that historical and structural similarities between the countries would serve as a basis for more horizontal and effective cooperation.

The goals of overcoming dependence on the North influenced counter-hegemonic formulations in international health relations. International health had begun to develop as a field of study in the 1960s, and several units dedicated to this subject were being established in US universities. These centers were dedicated to exploring aspects such as the occurrence of diseases in poor countries and their determinants, the effects of malnutrition, the internationalization of health achievements, comparisons between health policies and systems, and cultural elements related to the health/disease process in different locations. However, these US experts were shaping the field of international health studies from their own perspectives, assuming they knew what was best for the less-developed countries of the Global South (Godue, 1992).

Ferreira’s group sought to understand international health as a dimension of the social structure, drawing on Marxist perspectives and liberation ideals, and questioned the exaggerated belief in technology as part of international health promoted by developed countries. They opposed the superiority of biomedical knowledge and the programs to combat mosquitoes and diseases carried out by WHO and its regional agencies, which in the 1970s had proven ineffective while poor populations remained unserved.

When Ferreira arrived at PAHO, one of his colleagues in the Human Resources Division was the Argentine doctor and sociologist Juan César García, who from 1965 to 1968 had conducted an extensive investigation into the state of medical education in Latin America, which was published in 1972. This work, in which García analyzed professor profiles, student aspirations, and the characteristics of one hundred medical schools, had major repercussions among progressive intellectuals in the region and became a reference on the need to incorporate the social sciences into medical education. Prior to his death in 1984, García published a series of other works in which he used Marxist and Gramscian references to understand the influence of social structure on disease. His ideas included developing theoretical perspectives to understand health production and medical practice, analysis of how the biomedical perspective influenced scientific research, the shaping of schools of public health in Latin America and behavior among medical elites, as well as criticism of the Rockefeller Foundation’s role in disseminating a vision he considered authoritarian and imposing, for example (Nunes, 1994, 2013).

In addition to García, many other health workers who were part of the Latin American social medicine movement (which flourished as a counter-hegemonic movement in the 1970s and 1980s when Brazil and other countries in the region were under dictatorial regimes) were colleagues, employees or students of the programs coordinated by Ferreira. A notable figure among these was the Brazilian sanitarian Sergio Arouca, who served as a PAHO consultant in Nicaragua and in the 1980s became one of the main defenders of Brazilian health reform.

During his first four years in PAHO’s Human Resources Division, Ferreira served as regional consultant for medical education and promoted workshops with teachers from Latin American countries to discuss solutions for health systems, published articles on the integrated IDA approach, coordinated a program to lower the cost of textbooks for medical students in the region, edited the journal *Educación Médica y Salud* and offered consulting services to countries.

While still in this post, during a mission to Brazil in 1973 Ferreira conceived the Program for the Strategic Preparation of Health Personnel (Programa de Preparação Estratégica de Pessoal em Saúde, PPREPS), together with Ernani Braga and Carlos Vidal Layseca, a Peruvian professor of preventive medicine at the Universidad Cayetano Heredia and head of PAHO’s Human Resources area in Brazil. The PPREPS program operated in Brazil from 1976 to 1982 and established IDA centers in regions across Brazil, offering support for the regionalization that was being advocated at the time, and through these centers promoted significant expansion in training for professionals in various health fields. The format of the program and its legacies helped shape schools of public health and consolidate the Brazilian field of human resources in health (Pires-Alves, Paiva, 2006), serving as a model for Ferreira’s (1976) formulations on international health cooperation. The program was also one of the pillars in the development of progressive health thinking in Brazil, which served as the foundation for the Brazilian health reform movement during the following decade (Castro, 2008; Escorel, 1999).

As for the format of cooperation, it was an initiative anchored in planning, formulated and carried out by national personnel with international support (from PAHO), which inspired Ferreira to lay out the idea in an article published in the journal *Educación Médica y Salud* in 1976, in which he critically discussed health cooperation in the light of ideas from the “third world” countries movement and proposed an “ideal” model for health cooperation (Ferreira, 1976). In this article, Ferreira argued that “traditional technical assistance” was linked to the assumption that twentieth century Western technology was “the best model for all developing peoples” and that cooperation under this assumption moved in one single direction, resulting in the transfer of knowledge that was not suited to the needs of developing countries and generated a cycle of dependency (Ferreira, 1976, p.336).

In his opinion, if cooperation were directed towards the search for “native” solutions based on “local creativity and the adaptation of relevant knowledge,” the divisions between developed and underdeveloped could become less defined and horizontal cooperation would promote the confidence necessary for self-sufficiency. For this to happen, the projects had to be “absolutely flexible” and consider accommodating local resources from the outset, with the international agent acting as a facilitator in adapting successful international experiences to conditions similar to the local realities. He stressed that “no new technology is valid *a priori*” and should not be above the “potential creation of a native technology” (Ferreira, 1976, p.337-338).

In saying this, Ferreira did not reject technological development, but relativized its *a priori* value, promoting the idea of adaptation. His work was based on criticism of project models oriented towards results that could be measured in the short term and suggested that cooperation should be geared towards developing institutions, with a well-designed prior planning stage to accommodate risks (Ferreira, 1976, p.339). On this point, he argued that establishing networks of similar institutions would create a locus for exchanges that would facilitate development, and that this proposal was similar to the PPREPS format that was being developed in Brazil.

By the time he published this work, Ferreira had already been promoted to director of PAHO’s Human Resources Development Department, at which point his activities (which had previously focused only on medical and public health education) began to include other health professions. In this position, which he held for 21 years, he wrote several works on medical education and administrative organization models for teaching institutions based on experiences he was familiar with (like the Chinese strategy of barefoot doctors) and those he had helped implement (such as the organization of primary care in Cuba).

Meanwhile, reflections on cooperation models continued at PAHO. In 1985, the organization had set up an International Health Residency Program,^4^ which received ten professionals each year in Washington who acted as junior advisors in areas selected according to their professional activity or interest. The prerequisite was a completed master’s degree in public health and the capacity for reflection. In the late 1980s, the students and graduates of this program proposed a seminar entitled “International health: a field of study and professional practice,” which critically discussed the concept of international health.

Since the 1980s, orthodox liberal thinking had dominated international relations under the leadership of the US, Britain and the Bretton Woods institutions, and the movement for cooperation between developing countries had not flourished. By the end of the decade the Berlin Wall fell, symbolizing the defeat of socialism and opening the door to the breakdown of the USSR (which would take place in 1992); the era of globalization began under the hegemony of neoliberal thinking. The development of communications media and emergence of AIDS and other new diseases (as well as the re-emergence of old ones under the guise of new behaviors) drew attention to increasing interdependence between countries, a context which was also reflected in the views of this group of health professionals.

It was within this international context that the event proposed by the students in PAHO’s International Health Residency took place in Quebec, Canada, in March 1991, bringing together around forty participants from Latin America, the United States and Canada. The meeting focused on trying to overcome the classic approach to international health built on the intention of empowering the other or the different, based on the idea of an implicit North that held the knowledge. As part of this approach, developing countries (or social minorities in developed countries) emerged as a complete system, ignoring the relationships, specificities and subjectivities that developed within these systems and to a certain extent defined them. The practices carried out under this approach were consequently homogeneous and decontextualized responses imposed on heterogeneous realities and also focused on assistance, preventing the development or strengthening of scientific and technological capacity within underdeveloped countries.

To overcome this restricted focus, the seminar participants stated that the primary objective of a new approach to international cooperation in health would be to strengthen *self-capacity* for countries, replacing the idea of dependence with interdependence, which in turn would drive bilateral and multilateral measures to replace the center/periphery model. Ferreira was in charge of writing the final report of the meeting, along with Charles Godue, a Canadian doctor from the Community Health Department of the Maisonneuve-Rosemont Hospital in Montreal; Laura Nervi, an Argentine anthropologist serving as a consultant for PAHO’s Human Resources Program; and Maria Isabel Rodrigues, a Salvadoran physician and coordinator of PAHO’s Residency Program.

One participant in the seminar was Paulo Buss, who at the time was director of the National School of Public Health at Fiocruz; in his speech at the event, he described the world that was transforming at the start of the last decade of the twentieth century as a time when inequalities between and within nations were widening. He highlighted the consequences of the debt crisis for developing countries, the millions of people living in poverty, and injustice in the international division of production, which was being exacerbated by transnationalization of companies. He argued that the concept of international health was determined by understandings of the health/disease process and of how societies and international relations should be organized; that “biological reductionism” served as the underpinning of the idea that “tropical diseases” should be combated in underdeveloped countries, reproducing a notion of the “sick tropics” that was characteristic of late-nineteenth-century thinking. He also stated that from the viewpoint of international agencies and the main donors, the countries in need of aid appeared as a single unit without their own histories, cultures or scenarios. In his view, international health programs should incorporate the concepts and theoretical and methodological frameworks developed by Latin American social medicine, based on utopia and solidarity (Buss, 1992).

Buss and Ferreira had met in 1979 at a meeting in Brasilia, when the creation of the Brazilian Association of Collective Health (Associação Brasileira de Saúde Coletiva, ABRASCO) was being discussed. The year after the Quebec meeting, in 1992, Ferreira invited Paulo Buss to join a PAHO mission to the USSR at the invitation of that country’s authorities to learn about paramedic training initiatives. When Ferreira decided to retire from PAHO at the age of 62, Paulo Buss (then vice-president of Fiocruz) invited him to take over as the foundation’s International Cooperation coordinator; Ferreira returned to Brazil in 1996.

## Working at Fiocruz and the PAHO partnership, within the framework of Brazilian foreign policy

On his return, Ferreira spent a few months at the International Cooperation Office at Fiocruz, an area that was mainly dedicated to the bureaucratic processes of organizing exchanges and other activities abroad for researchers. Some months later, Paulo Buss left his position as vice-president of Fiocruz and returned to work at ENSP. Ferreira accompanied him and began activities at the school he helped to create thirty years earlier. In 1998 Buss was elected director of ENSP, and Ferreira became his right-hand man for international affairs. In the office of Fiocruz’s vice-presidency and at ENSP, Ferreira’s activities focused on promoting academic exchanges, establishing agreements with internationally recognized institutions such as the US National Institutes of Health and the Canadian government, which were responsible for introducing new fields of knowledge to the school and the foundation (Kastrup, 2015).

In 2000 Paulo Buss was elected president of Fiocruz, and Ferreira took over as the foundation’s international advisor. Buss had a special interest in Fiocruz occupying a relevant position in global health and found the context favorable, as discussions around the Millennium Development Goals (MDGs) and the entry of emerging countries like Brazil into the dispute for power in international relations drove a resumption in cooperation between developing countries (Cabana, 2014).

The main means of achieving the MDGs was international cooperation, and for this the practice had to work well. However, there were many critiques of the models adopted since the end of Second World War: for example, cooperation had been too ideologized and driven by the interests of the countries involved in the Cold War. Many analysts argued that the projects were generally ineffective because they were based on solutions developed in settings different from the realities in which they were implemented, or that the aid chain had become an end in itself and neglected the political choices of the recipient countries, or that the requirements and various accountability models adopted by donor countries created a huge bureaucracy for the administrative structures of the countries that needed aid (Foster, 2000; Sogge, 2004). This criticism strengthened the discourse around SSC, which was intended to be a more effective model. Within this context, developed countries began to hold debates on how to improve the practice of international cooperation.

Throughout the first decade of the twenty-first century, the Organization for Economic Co-operation and Development, which brought together these main countries, held a series of meetings (in Rome in 2003, Paris in 2005, Accra in 2008, and Busan in 2011) that sought solutions to the problem of ineffective cooperation and culminated in recommendations: actions should be coordinated within the territories of aid recipients; donors should promote actions that are aligned with the national policies of recipient countries; the different activities promoted by different donors should be harmonized to avoid fragmentation.

Within this environment of debate about the practice of international aid, emerging countries found a space to revive some of the NAM perspectives that had lost strength in previous decades and began to criticize the cooperation promoted by Northern countries, claiming that their activities were more effective because they better understood the challenges of the poorest nations given that, despite being growing economies, they still faced similar challenges. They stated that their cooperation was based on mutual aid, without imposing solutions or conditionalities like the purchase of products or adoption of policies suggested by donor countries. They sought to differentiate themselves from more developed countries by avoiding terms like donor, recipient, assistance and aid, and preferred to refer to partnership and cooperation (Esteves et al., 2011).

It was by taking advantage of this context that Brazil developed the foreign policy strategies that would be adopted throughout the first decade of the twenty-first century. Between 2003 and 2010, Brazilian foreign policy aimed to establish the nation as an emerging power and seek a leadership position in the Global South through a strategy of diversifying its partnerships, drawing closer to other developing countries and focusing on SSC as a way to expand its strength and prestige in international relations (Vigevani, Cepaluni, 2007; Saraiva, 2007, 2013). At the same time, health was becoming an interesting national issue for foreign policies: on the one hand, because of the threat that the spread of diseases posed to national security, and on the other because of movements that sought to shape health as an object of human solidarity and social justice (Fidler, 2007).

Internally, within the sector, Lula’s election as president in 2003 represented a moment of optimism for progressive health workers who had participated in the movements for Latin American social medicine and Brazilian health reform at different times, in various ways. The rise of a progressively-leaning government meant that sanitarians from these movements came to occupy positions in the Ministry of Health, such as José Saraiva Felipe, a doctor from Minas Gerais who had participated in an important health reform project in the city of Montes Claros in that same state and took over the Brazilian Ministry of Health in 2005. Felipe invited Francisco Eduardo Campos (another physician from Minas Gerais, who had been Ferreira’s advisor at PAHO from 1989 to 1996) to lead the ministry’s Department of Work Management and Health Education (Secretária de Gestão de Trabalho e da Educação em Saúde, SGTES). One of Campos’ main projects within this department was to redirect the teaching of health professions towards an emphasis on primary care, and he invited Ferreira to participate as an advisor to the Ministry of Health on this subject.^5^ In this way, nine years after arriving in Brazil, Ferreira began to combine his work in the International Cooperation Office at Fiocruz with the debate on teaching in the health professions, two roles he dedicated his entire career to.

With Campos at the helm of SGTES, a cooperation project was developed with PAHO known as TC-41, which would become one of Brazilian foreign policy’s pillars for cooperation in health between 2005 and 2015. At that time, because of the scale of its institutional activity Fiocruz became the main institution advising and executing health cooperation within the framework of this foreign policy, and this program was coordinated by José Roberto Ferreira. The partnership with Paulo Buss, who had been president of Fiocruz since 2000, was intense. Buss mainly dedicated himself to health diplomacy, taking part in various boards at WHO, PAHO, the Community of Portuguese Language Countries, and later the Union of South American Nations; Ferreira, meanwhile, was in charge of putting cooperation into practice, discussing the scope of projects with leaders of partnering countries and technicians from the Ministry of Health and the Brazilian Cooperation Agency within the Brazilian Ministry of Foreign Affairs, bringing together and guiding Fiocruz technicians and researchers who carried out activities abroad.

The experience surrounding this movement was systematized in the article that introduced the notion of SSSC; its main author, Celia Maria de Almeida, was the director of the Fiocruz office in Maputo, Mozambique at that time. Celia is a physician, a retired ENSP researcher, and had dedicated part of her professional life to working in Mozambique, with research focused on the study of health sector reforms in different countries and health systems from a comparative perspective.

The article that introduced SSSC, with a list of authors including Almeida and Buss (Almeida et al., 2010), illustrated the interconnections between thinking on international sanitarism (which had been cultivated at Fiocruz since at least the 1990s) and the government policy of utilizing health cooperation as an element of foreign policy. The text did not represent an attempt at *a priori* theorizing, but presents an ongoing process organized on the theoretical foundations that the authors systematize in the text. The paper began with a critical historical review of international cooperation as well as international cooperation in health that sought to explain how international health activities were conditioned by interests and circumstances that have not helped aid improve the health conditions of the populations that require it. The authors attributed this reality to the neoliberal hegemony that solidified globally from the 1980s onward, with structural adjustment policies prescribed by the World Bank, the AIDS epidemic that overloaded health systems, migration of health professionals from poorer nations to the countries of the North, as well as the “traditional” model of North-South health aid geared towards combating specific diseases.

Based on these arguments, the authors defended a way to think about cooperation in health based on the precepts of SSC, with the central objective of structuring health systems. The model was centered on developing countries’ internal capacities and focused on institutions considered structural for health systems, such as ministries of health, schools of public health, national institutes of health, and technical schools. For the authors, this concept was based on building capacities for development, which was innovative in that it integrated human resources training and institutional development and proposed taking advantage of the country’s own capacities and resources (Almeida et al., 2010, p.28).

In short, SSSC incorporated notions of how to cooperate, based on the ideals of SSC, together with what should be done in terms of cooperation in the area of health; in other words, it combined the proposal to build partnerships, exchange experiences, and share results and learning with the understanding that capacities should be developed by mobilizing local knowledge, based on each place’s individual reality, so that recipient countries can develop autonomous agendas for their health development.

Criticism of international health models is more explicitly present in another article by Buss and Ferreira, published in the same issue of the journal that included the text on structuring cooperation. In that text, the authors (Buss, Ferreira, 2010a) described the dominant model of international cooperation, pointing out that developed countries impose their own worldviews on less-developed countries. They also emphasized (as did Ferreira’s 1976 article) the difference between assistance and cooperation, and by incorporating the principles defined in the debates on the effectiveness of international aid they disapproved of the lack of coordination and overlapping of projects in poor countries. They updated the critique of vertical agendas, arguing that the main players in global health adopted one-way communication, ignoring processes already in place in the countries where the projects were carried out.

Vertical programs do not help to strengthen the system as a whole; on the contrary, they lead to its fragmentation and weakness, either by recruiting the best personnel available in the country, or by concentrating on certain areas and abandoning other priority areas (Buss, Ferreira, 2010a, p.96).

In this way, the authors returned to a dilemma in global health fraught with ideological concepts that permeated international health: the verticalized fight against disease versus prevention and the structuring of health care (Cueto, 2004), and accused public/private health partnerships of providing traditional care (understood to be ineffective). This perspective reveals the influence of a critical perspective present in the Latin American social medicine movement.

During the early 2010s, as deputy coordinator of the Oswaldo Cruz Foundation’s Center for International Relations in Health, Ferreira published several articles on the foundation’s experiences with SSC in health, mainly with the Union of South American Nations and the Community of Portuguese Language Countries (Buss, Ferreira, 2010b, 2011, 2012, 2017; Buss, Ferreira, Hoirisch, 2011; Ferreira et al., 2016; Ferreira, Fonseca, 2017).

This body of work characterized the final stage in Ferreira’s professional career, marked by his defense of the concept of structuring South-South cooperation and his quest to demonstrate its application and development in Brazil’s principal spaces in SSC.

While more research is still needed on Brazil’s role in international health cooperation from 2005 onwards to compare discourse and practice (Russo, Cabral, Ferrinho, 2013), the last stage of Ferreira’s professional career is marked by an effort to propagate the conceptual frameworks developed throughout his career that guided it, and which found political space for implementation through projects and regional alliances within the framework of Brazilian foreign policy. Although the strategy was gradually discontinued during the 2010s, the group of projects which were carried out and the shaping of a group of professionals at Fiocruz simultaneously dedicated to international technical cooperation and to developing this concept through academic debates and publications made it possible to disseminate the concept of structuring South-South cooperation within the institution itself and in other forums in the Brazilian Ministry of Health and in Brazil’s health diplomacy (Kastrup, 2023).

## Final considerations

We have described the role of José Roberto Ferreira’s trajectory in constructing the concept of structuring South-South cooperation, which became a guiding principle for Brazil’s international technical cooperation activities in health during the first decade of the 2000s. The start of this trajectory was marked by concern with building medical schools that incorporated the reality of basic health services as part of training for medical professionals, which led Ferreira to a position at PAHO, where while being in charge of regional cooperation and inspired by the movement to bring developing countries closer together, he shaped a concept of technical health cooperation intended to overcome dependence in the countries receiving these actions. This perspective began from the notion of using local realities and native knowledge to build health solutions. From this perspective, the role of the international agent (or the country offering its supposed expertise) would be to actively listen to and strengthen local staff and authorities.

These ideas of an emancipatory cooperation model developed at PAHO’s Human Resources Division in Washington are closely related to the Latin American social medicine movement, which was also promoted there and proposed training doctors and health workers who were sensitive to local and social realities of populations, leading to the construction of an emancipatory concept of international health.

From our perspective, these ideas were transposed to global health in the midst of a progressive government in Brazil that valued SSC and health as instruments of foreign policy. Ferreira’s position at Fiocruz and the foundation’s role in developing this foreign policy made it possible to transpose this concept and formulate the idea of structuring South-South cooperation, which also played a fundamental role in the ideas and experiences of the other authors of the article that described this cooperation. The proposal to strengthen institutions and train human resources as a means of promoting the autonomy and sustainability of health systems in recipient countries indicates the central role played by José Roberto Ferreira.

## Data Availability

The data is not stored in a repository.

## NOTES

1 The article was authored by Celia de Almeida, who in 2010 was head of Fiocruz Africa office; Paulo Buss, head of the Fiocruz Center for International Relations in Health (Centro de Relações Internacionais em Saúde, CRIS); Luís Eduardo Fonseca, a physician and technician at the CRIS; Rodrigo Pires de Campos of the Department of International Relations at the University of Brasilia; and José Roberto Ferreira, deputy director of the CRIS at that time.

2 During the first phase of his career, Ferreira published 18 scientific works on surgery that include articles, publications and conferences.

3 In 1955 and 1956, PAHO held two historic seminars in Viña del Mar, Chile and Tehuacán, Mexico, which are considered milestones in the introduction of the theme of preventive medicine in Latin America.

4 The program continues to this day as the Leaders in International Health Program.

5 Ferreira was part of the Pró-Saúde project.
