# Supplementary material for: José Roberto Ferreira: an intermediary between international health and global health, 1959-2019
Source: Hist Cienc Saude Manguinhos. 2025 May 2;32:e2025017. doi: 10.1590/S0104-59702025000100017en (PMC12052331; doi:10.1590/S0104-59702025000100017en)
Supplement: Translation

# Translation: José Roberto Ferreira: um intermediário entre a saúde internacional e a saúde global, 1959-2019

**DOI:** 10.1590/S0104-59702025000100017

**Authors:** Erica Kastrup, Analice Pinto Braga, Gabriel Lopes

**Affiliations:** i Analista de Cooperação Internacional, Centro de Relações Internacionais em Saúde/Fiocruz. Rio de Janeiro – RJ – Brasil. erica.kastrup@fiocruz.br; ii Assessora de Relações Internacionais, Fiocruz. Rio de Janeiro – RJ – Brasil. analicepbraga@gmail.com; iii Pesquisador em estágio pós-doutoral, Casa de Oswaldo Cruz/Fiocruz. Rio de Janeiro – RJ – Brasil. lopes-gabriel@hotmail.com

**Keywords:** Saúde global, Saúde internacional, Cooperação Sul-Sul, Educação em saúde, José Roberto Ferreira (1934-2019

## Abstract

O artigo apresenta a contribuição de José Roberto Ferreira para as discussões, nos âmbitos nacional e internacional, a respeito da formação de profissionais da saúde e de suas contribuições para a construção de uma proposta crítica para a cooperação Sul-Sul em saúde. Argumenta-se que Ferreira defendia ser a educação em saúde, desenvolvida com base em realidades particulares de países menos desenvolvidos, um caminho para a emancipação. Inicialmente apresentamos a formação e os primeiros trabalhos de Ferreira no campo da educação (1959-1969); as suas experiências internacionais e os contatos com figuras importantes da medicina social latino-americana (1969-1996); e, finalmente, o seu trabalho na Fiocruz no âmbito da política externa para desenvolvimento da cooperação estruturante em saúde (1996-2019).

A saúde ganhou importante espaço na política externa brasileira a partir do início do século XXI, e, no caso específico do governo de Luiz Inácio Lula da Silva, as ações nesse campo representaram um dos principais instrumentos de uma política externa que pretendia aproximar o Brasil de outros países em desenvolvimento no Sul global. A ideia era conformar o Brasil como potência emergente capaz de apoiar o desenvolvimento de outros países por meio da cooperação técnica e do intercâmbio de experiências sobre políticas públicas nacionais bem-sucedidas, com destaque para o Sistema Único de Saúde (SUS) (Almeida et al., 2023; Buss, 2018; Cueto, Lopes, 2023). Nesse contexto, um grupo de sanitaristas brasileiros publicou um artigo no qual apresentaram o conceito de cooperação Sul-Sul estruturante em saúde (daqui em diante, CSSE), que propôs temas e modelos de relação que norteassem a ação sanitária do Brasil internacionalmente (Almeida et al., 2010).

A ideia de CSSE traz em seu bojo a crítica de que, historicamente, a cooperação internacional em saúde havia sido ineficiente por ser formulada externamente de onde seria aplicada, gerando dissonância entre as ações e as reais necessidades dos países que seriam os beneficiários delas. A partir desse entendimento, propunha que a cooperação brasileira fosse orientada pelas demandas apresentadas, estabelecendo parcerias e respeitando as racionalidades específicas dos parceiros. Para isso, os beneficiários deveriam participar ativamente no desenho dos projetos, evitando a transferência passiva de conhecimentos e promovendo o empoderamento de autoridades públicas locais com relação à intervenção que seria realizada em seu território. Entre os temas centrais dessa cooperação em saúde estavam a formação de recursos humanos e o fortalecimento institucional, de modo que os resultados dos programas e projetos resultassem na estruturação de instituições que fossem pilares dos sistemas de saúde locais, que seriam então capazes de conduzir processos autônomos de desenvolvimento por meio da saúde (Almeida et al., 2010; Ferreira et al., 2016).

Esse modelo de cooperação dialogava com a agenda da restruturação dos sistemas de saúde que emergia na primeira década dos anos 2000 associada à ideia de eficácia da ajuda para o desenvolvimento. O artigo de Almeida et al. (2010) aponta que as ideias sobre o conceito de cooperação estruturante foram oriundas do processo de desenvolvimento dos projetos que estavam sendo oferecidos pelo Brasil a outros países do Sul global no marco da proposta da política externa assertiva e altiva adotada durante o governo de Lula da Silva (2003-2010). De fato, esse foi um momento propício para que o governo pudesse empregar a proposta de cooperação estruturante e disseminar sua matriz ideológica. Tal concepção da cooperação internacional foi engendrada pelo médico brasileiro José Roberto Ferreira, a partir de suas experiências pessoais e de uma teia de relações que permearam sua carreira, que perpassou a saúde internacional e a saúde global.

Este artigo, nesse sentido, argumenta que Ferreira teve um papel essencial para a fundação das bases do que ficou conhecido como “cooperação Sul-Sul estruturante”, conceito sistematizado e desenvolvido como parte de uma agenda da política externa brasileira para a saúde e publicado na *Revista Eletrônica de Comunicação, Informação & Inovação em Saúde – Reciis*, do Instituto de Comunicação e Informação Científica e Tecnológica em Saúde da Fundação Oswaldo Cruz (Fiocruz), em março de 2010, artigo do qual Ferreira foi um dos autores (Almeida et al., 2010).^1^

Para delinear o nosso argumento, vamos apontar três momentos fundamentais da carreira de Ferreira. O primeiro, de 1959 a 1969, retrata o início do trabalho do jovem médico, que associava pesquisas no campo da cirurgia cardíaca ao desenvolvimento de projetos de escolas de medicina que aproximassem o ensino dos serviços de saúde. O segundo refere-se a seu trabalho na Organização Pan-Americana da Saúde (Opas), de 1969 a 1996, quando conviveu com nomes importantes do movimento da medicina social latino-americana e, desde uma perspectiva internacional, passou a defender que a educação em saúde, desenvolvida com base em realidades particulares de países menos desenvolvidos, funcionaria como um meio de emancipação desses países de um sistema de cooperação internacional que reproduzia assimetrias. Nesse sentido, construiu uma perspectiva sulista, que associava a prática da cooperação internacional em saúde a ideias de emancipação. O terceiro momento, de 1996 a 2019, compreende os anos de trabalho na Fundação Oswaldo Cruz, na qual pôde colocar tais ideias em prática por meio de projetos de cooperação Sul-Sul (daqui em diante, CSS) desenvolvidos pelo governo brasileiro.

José Roberto Ferreira faleceu em 25 de dezembro de 2019. As fontes utilizadas para a reconstrução de sua trajetória incluem um relato de vida do próprio, manuscrito que foi concedido por sua filha em 2020. A análise desse material foi complementada com as entrevistas dadas por ele ao Observatório História e Saúde da Casa de Oswaldo Cruz e com artigos, livros publicados e outros trabalhos acadêmicos que discutem a cooperação em saúde oferecida pelo Brasil, que serão mencionados ao longo do texto.

## O início da carreira e o interesse pelo desenvolvimento institucional

José Roberto Ferreira iniciou sua carreira como médico no fim dos anos 1950, quando se dedicou à cirurgia cardíaca no hospital de Ipanema. Seu pai era um renomado especialista em tuberculose e membro titular da Academia Nacional de Medicina, o que abriu portas para que Ferreira fosse bem relacionado com a elite da sociedade médica do Rio de Janeiro dos anos 1950 e 1960. Interessado no desenvolvimento das tecnologias médicas e na pesquisa acadêmica,^2^ foi a partir de contatos de seu pai que Ferreira se tornou redator do *Jornal Brasileiro de Medicina* em 1959, dois anos depois de sua formatura pela Universidade do Brasil, no Rio de Janeiro. Interessado na modernização da cirurgia, viajou aos EUA como assistente de Lucio Galvão, um cirurgião emérito da Academia de Medicina, para pesquisar a técnica de circulação extracorpórea que ele estava trazendo para o Brasil.

Sua iniciação no campo da gestão e administração do ensino médico se deu justamente por intermédio de Galvão, que o convidou para substituí-lo em um grupo de trabalho criado para elaborar as diretrizes básicas para a reforma da Universidade do Brasil. Segundo o próprio Ferreira, o cirurgião emérito não tinha nenhum interesse em atividades administrativas, ao contrário do jovem assistente, motivo pelo qual Galvão solicitou sua própria substituição. Nesse grupo, Ferreira criou uma teia de relações que vieram a impulsionar seu interesse pelo desenvolvimento de instituições de ensino da medicina.

Um dos membros do grupo era Ernani Braga, que trabalhava em projeto da Fundação Rockefeller junto à Coordenação de Aperfeiçoamento de Pessoal de Nível Superior (Capes) no Brasil, por meio do qual se dedicava ao fomento à formação de médicos e enfermeiros no país e colaborava com outros países latino-americanos nos quais a Rockefeller desenvolvia o mesmo projeto. Em 1962, foi criada a Federação Pan-Americana de Faculdades e Escolas de Medicina, e Ernani foi convidado para ser diretor-executivo da nova organização. Abriu-se um escritório no Rio de Janeiro, em um espaço cedido na sede da Opas no Brasil, e Braga convidou Ferreira para ser seu assistente. A federação, sob o patrocínio da Opas, da Fundação Rockefeller, e de outras fundações filantrópicas norte-americanas, tinha como uma de suas atribuições fomentar a conformação de redes nacionais de escolas de medicina, o que se consolidou no Brasil com a fundação da Associação Brasileira de Educação Médica no ano seguinte, para a qual Ferreira foi designado diretor-executivo. Naquele momento, a Opas também fundava uma área dedicada à formação dos recursos humanos para a saúde no Brasil.

Outro colega do grupo de trabalho para a reforma da Universidade do Brasil era Durmeval Trigueiro Mendes, então diretor de ensino superior do Ministério da Educação e Cultura (MEC), que convidou Ferreira para secretariar a Comissão de Planejamento da Formação de Médicos (CPFM), grupo que estava sendo constituído para essa tarefa no âmbito do MEC. Ferreira aceitou a posição, que consistia em analisar as solicitações de abertura de novas escolas de medicina, funcionando como uma assessoria a um movimento de expansão do ensino médico que ocorria no Brasil ao longo dos anos 1960.

Entre 1965 e 1970, foi autorizado o funcionamento de 33 novas escolas de medicina com subsídios governamentais (Haddad et al., 2010). Ao mesmo tempo, no plano regional, a Opas vinha discutindo a medicina preventiva, desde meados dos anos 1950,^3^ com a proposta de incluir no currículo das faculdades médicas uma disciplina que articulasse o ensino das ciências biológicas ao das ciências sociais. Esse movimento pode ser compreendido como um processo de resistência ao avanço do modelo biomédico na assistência à saúde, e, ao longo dos anos 1960, foi gradativamente sendo difundido no Brasil com a criação de núcleos e departamentos de medicina preventiva no ensino universitário (Arouca, 2003; Escorel, 1999).

Na CPFM, Ferreira fazia recomendações e adequações nas propostas, e redigia projetos de estrutura e funcionamento de escolas de medicina. Esse trabalho, que ocorria concomitantemente às suas atividades na Associação Brasileira de Educação Médica, potencializou o intercâmbio entre as escolas de medicina brasileiras. Entre as escolas que estavam sendo criadas, estava a Escola Nacional de Saúde Pública da Fundação Oswaldo Cruz, cujos estatutos foram elaborados por Ferreira em 1965.

No ano seguinte, convidado para participar da elaboração da nova Escola de Medicina da Universidade de Brasília (UnB), Ferreira foi um dos responsáveis pela construção de um projeto que foi considerado inovador por incorporar também o ensino da enfermagem e da odontologia às disciplinas básicas comuns, orientando os currículos por eixos integrados de ensino biomédico, saúde pública e prática clínica, o que delineava um novo perfil de curso baseado na integração do ensino com serviços médicos comunitários.

O projeto fundamentava-se na ideia de Integração Docente-Assistencial (IDA), que foi uma abordagem da educação em saúde que preconizava a necessidade de maior integração entre a formação e a prática nos serviços de saúde (Pires-Alves et al., 2010; Pires-Alves, Paiva, Hochman, 2008). Em linhas gerais, essa abordagem promovia o ensino a partir de uma integração multiprofissional com ênfase nos serviços de atenção básica e proporcionava a interdisciplinaridade no ensino médico. A IDA e a medicina preventiva, sob perspectivas distintas, defendiam ser importante a ampliação do olhar do médico para perspectivas mais holísticas na abordagem do processo saúde-doença.

Mais tarde, nesse mesmo ano, Ferreira foi convidado pelo então reitor da UnB para ocupar o cargo de vice-reitor. Entusiasmado com o projeto de universidade criado por Darcy Ribeiro com o aval do então ministro da Educação, que Ferreira havia conhecido na antiga comissão do MEC, aceitou o convite e mudou-se para a nova capital federal. Àquela altura, Ernani Braga havia se mudado para Genebra, para ocupar o cargo de diretor do Departamento de Desenvolvimento de Recursos Humanos da Organização Mundial da Saúde (OMS), então liderada pelo brasileiro Marcolino Candau, seu amigo de longa data. Na OMS, Ernani passou a promover a Escola de Ciências da Saúde da UnB como um modelo, o que difundiu o nome de José Roberto Ferreira como especialista na conformação de escolas médicas e de saúde pública, além de ter gerado um movimento de visitas de autoridades internacionais ao *campus* da universidade. Em 1967, Braga convidou Ferreira, junto com um consultor canadense, para assessorar a implementação de uma faculdade de medicina na República dos Camarões, ex-colônia francesa e inglesa que havia conquistado a independência poucos anos antes, experiência que consistiu na iniciação de Ferreira no campo da cooperação internacional.

Em seu primeiro trabalho em território africano, como consultor da OMS, Ferreira pensou inicialmente não ser possível a criação de um curso universitário em condições tão precárias, mas foi dissuadido pelo próprio Marcolino Candau, que indicou que era necessário encontrar um modelo capaz de responder àquele desafio.

Ferreira elaborou a proposta de criação do curso, que foi aprovada pela OMS e financiada pelo Banco Mundial em 1968, momento em que se intensificavam as manifestações em oposição à ditadura civil-militar no Brasil, em especial contra o ato institucional n.5 (AI5), decretado pelo general Artur da Costa e Silva, em dezembro daquele ano. Os cercos aos universitários e à própria universidade fizeram com que Ferreira renunciasse ao cargo de vice-reitor da UnB.

Segundo o próprio, a repercussão do seu trabalho em Camarões na sede da OMS, em Genebra, foi o que motivou o convite do então diretor da Opas, Abraham Horwitz, para que ele fosse ocupar um cargo de consultor regional para educação médica na sede da organização, em Washington.

## Saúde internacional, cooperação Sul-Sul e a ideia de foco no “autóctone”

José Roberto chegou à sede da Opas, na capital dos EUA, em janeiro de 1969. A partir daí, seu exercício profissional passou a aliar a preocupação com os modelos de cooperação em saúde aos princípios progressistas da saúde pública, que adquiriram características particulares nas Américas e conformaram o que ficou conhecido como medicina social latino-americana (Nunes, 2013; Tajer, 2003). Desde a sua criação, a Opas vinha se dedicando a discutir os perfis de atuação e as carreiras de médicos e outros profissionais de saúde na região e, naquele momento, conformava-se, na Divisão de Recursos Humanos na sede da organização na capital norte-americana, um núcleo de pensamento que buscava associar conhecimentos em ciências sociais à formação de pessoal de saúde. Esse núcleo, nos anos seguintes, promoveu debates, desenvolveu ideias, teses, programas de ensino e projetos de cooperação orientados pela visão social da saúde e se configurou como um núcleo de tensão à hegemonia do modelo biomédico na saúde internacional.

As ideias da medicina social latino-americana contrapunham-se às propostas de se combater e até mesmo erradicar doenças sem a realização de mudança nas condições sociais em que as pessoas viviam. Nesse sentido, estavam imbuídas de um ideal de transformação social que se traduzia na defesa do acesso equitativo aos serviços de saúde, na provisão de serviços como uma responsabilidade do Estado e na importância das ciências sociais e da política na saúde pública (Nunes, 2013, 2015; Tajer, 2003). O movimento da medicina social latino-americana não se restringiu à área de recursos humanos, mas ali teve suas origens, principais formulações e sustentabilidade por meio de programas de ensino e pesquisa que propunham uma delimitação conceitual da saúde pública que se ancorava na determinação social, econômica e política do processo saúde-doença (Castro, Germano, 2010).

Essas ideias sobre organização de serviços, educação em saúde e sobre como promover a cooperação entre países floresceram na América Latina nos anos 1970 com influência de movimentos sociais e políticos que ocorriam em outros setores. A década de 1970 foi frutífera para o movimento de cooperação entre os países do chamado “terceiro mundo”, que começaram um processo de aproximação entre si nos anos 1950, quando do início dos processos de independência na Ásia e na África, e que se desenvolveu nos anos 1960 a partir de uma lógica de distanciamento do conflito ideológico Leste-Oeste e de contestação das relações de dominação do Norte sobre o Sul e do colonialismo.

Naquele momento, à medida que o processo de descolonização se intensificava, esse movimento se ampliava, ancorado na ideia de solidariedade entre as nações da periferia e de luta contra as injustiças que estruturavam o sistema internacional de países, e foi denominado Movimento dos Países Não Alinhados (MNA). Esses países queriam manter a soberania e a autodeterminação e organizar alianças de modo a diminuir a dependência dos Estados industrializados, buscando soluções compartilhadas e baseadas em características próprias, o que deu origem a uma agenda terceiro-mundista que se desenvolveria nos anos seguintes de maneira marginal à Guerra Fria (Almeida, 2017; Pereira, Medeiros, 2015; Pino, 2014).

Esse movimento se fundava em críticas sobre os modelos de cooperação Norte-Sul tidos como unidirecionais, assistencialistas, ineficazes e dirigidos por interesses e afirmava a Cooperação Técnica entre Países em Desenvolvimento como um instrumento da autonomia dos países menos desenvolvidos e como uma prática complementar à cooperação Norte-Sul. A ideia central era que as similaridades históricas e estruturais entre os países serviriam de base para uma cooperação mais horizontal e mais eficaz.

Os ideais de superação da dependência com relação ao Norte influenciaram formulações contra-hegemônicas nas relações internacionais em saúde. Nos anos 1960, a saúde internacional havia começado a se desenvolver como campo de estudos e diversas unidades dedicadas ao tema estavam sendo criadas em universidades norte-americanas. Esses núcleos se dedicavam a explorar aspectos como a ocorrência de doenças em países carentes e suas determinações, os efeitos da desnutrição, a internacionalização dos feitos sanitários, as comparações entre políticas e sistemas de saúde, os elementos culturais relacionados ao processo saúde-doença em diferentes localidades, entre outros. No entanto, esses especialistas norte-americanos estavam moldando o campo de estudos da saúde internacional seguindo perspectivas próprias, presumindo que sabiam o que era melhor para os países menos desenvolvidos do Sul global (Godue, 1992).

O grupo do qual Ferreira fazia parte buscava compreender a saúde internacional como uma dimensão da estrutura social valendo-se de perspectivas marxistas e ideais de libertação, e questionava a crença exagerada na tecnologia promovida pelos países desenvolvidos na saúde internacional. Eles estavam contra a superioridade do saber biomédico e os programas de combate a mosquitos e doenças levados a cabo pela OMS e as suas agências regionais, que, nos anos 1970, tinham se provado ineficazes, ao mesmo tempo que as populações pobres continuavam desassistidas.

Quando Ferreira chegou à Opas, um de seus colegas na Divisão de Recursos Humanos era o médico e sociólogo argentino Juan César García, que, entre 1965 e 1968, conduzira uma ampla investigação sobre a situação da educação médica na América Latina, publicada em 1972. Esse trabalho, no qual Garcia analisa o perfil de docentes, aspirações de discentes e características de cem escolas de medicina, teve grande repercussão entre intelectuais progressistas na região e conformou-se como uma referência sobre a necessidade de se incorporar as ciências sociais na educação médica. Garcia publicou uma série de outros trabalhos, até seu falecimento, em 1984, nos quais se utilizou de referências marxistas e gramscianas para compreender a influência da estrutura social na determinação das doenças. Suas ideias abrangem, por exemplo, a elaboração de perspectivas teóricas para se compreender a produção de saúde e a prática médica, a análise da influência da perspectiva biomédica na investigação científica e na conformação de escolas de saúde pública na América Latina, a análise do comportamento das elites médicas e a crítica à atuação da Fundação Rockefeller na disseminação de uma visão que ele considerava autoritária e impositiva (Nunes, 1994, 2013).

Além de Garcia, muitos outros sanitaristas que integraram o movimento da medicina social latino-americana, que floresceu como um movimento contra-hegemônico nas décadas de 1970 e 1980 – quando o Brasil e outros países da região viviam sob regimes ditatoriais –, foram colegas, funcionários ou alunos dos programas coordenados por Ferreira. Entre esses, vale a pena citar o sanitarista brasileiro Sergio Arouca, que foi consultor da Opas na Nicarágua e que, nos anos 1980, se tornou um dos principais articuladores da reforma sanitária brasileira.

Nos primeiros quatro anos na Divisão de Recursos Humanos da Opas, Ferreira assumiu o cargo de consultor regional para educação médica e promoveu oficinas com docentes de países latino-americanos para discutir soluções para os sistemas de saúde, publicou artigos sobre abordagem da IDA, coordenou um programa que buscava baratear o custo de livros para estudantes de medicina na região, editou a revista *Educación Médica y Salud* e ofereceu consultorias a países.

Ainda nesse posto, em 1973, durante uma missão ao Brasil, Ferreira concebeu o Programa de Preparação Estratégica de Pessoal em Saúde (Ppreps), junto com Ernani Braga e Carlos Vidal Layseca, um professor peruano de medicina preventiva da Universidad Cayetano Heredia e chefe da área de Recursos Humanos da Opas no Brasil. O Ppreps, que funcionou no Brasil entre os anos de 1976 e 1982, tinha como estratégia a implantação de núcleos de IDA em regiões do Brasil, oferecendo apoio à regionalização que se preconizava naquele momento, tendo promovido uma grande expansão na formação de profissionais em diversos campos da saúde por meio desses núcleos. O formato do programa e os seus legados contribuíram para a conformação de escolas de saúde pública e para a consolidação do campo de recursos humanos em saúde no Brasil (Pires-Alves, Paiva, 2006), e serviram de modelo para as formulações de Ferreira (1976) sobre a cooperação internacional em saúde. Além disso, o programa foi um dos pilares do desenvolvimento do pensamento progressista em saúde no Brasil, que ancoraram o movimento pela reforma sanitária brasileira na década seguinte (Castro, 2008; Escorel, 1999).

No que tange ao formato da cooperação, era uma iniciativa ancorada no planejamento, formulada e executada por pessoal nacional com apoio do agente internacional – a Opas –, o que inspirou que Ferreira formatasse a ideia em um artigo publicado na revista *Educación Médica y Salud*, em 1976, no qual discutiu criticamente a cooperação em saúde à luz das ideias do movimento dos países do “terceiro mundo” e propôs um modelo “ideal” para a cooperação em saúde (Ferreira, 1976). No artigo, Ferreira argumentava que a “assistência técnica tradicional” estava vinculada ao pressuposto de que a tecnologia ocidental do século XX era “o melhor modelo para todos os povos em desenvolvimento”, e a cooperação sob esse pressuposto se dava por uma via unidirecional, o que resultava em transferência de conhecimentos que não se adequavam às necessidades dos países em desenvolvimento e gerava um ciclo de dependência (Ferreira, 1976, p.336).

Para ele, se a cooperação fosse direcionada para a busca de soluções “autóctones”, baseadas na “criatividade local e na adaptação de conhecimentos pertinentes”, as fronteiras entre desenvolvido e subdesenvolvido poderiam ser diluídas, e a cooperação, horizontal, promoveria a autoconfiança necessária à autossuficiência. Para isso, era preciso que os projetos fossem “absolutamente flexíveis” e que desde o início considerassem a acomodação de recursos locais, em que o agente internacional funcionaria como um facilitador da adaptação de experiências internacionais bem-sucedidas em condições semelhantes às realidades locais. Destacava que “nenhuma tecnologia nova é válida *a priori*”, não devendo estar acima da “potencial criação de uma tecnologia autóctone” (Ferreira, 1976, p.337-338).

Com isso, Ferreira não rechaçava o desenvolvimento tecnológico, mas relativizava sua valoração *a priori*, advogando a ideia de adaptação. Seu trabalho se fundava na crítica a modelos de projetos orientados por resultados mensuráveis no curto prazo e sugeria que a cooperação deveria ser voltada para o desenvolvimento de instituições, com uma etapa prévia de planejamento bem elaborada para a acomodação de riscos (Ferreira, 1976, p.339). Nesse ponto, defendeu que a criação de redes de instituições semelhantes configuraria um lócus de intercâmbios que facilitariam o desenvolvimento e que a proposta era semelhante ao formato do Ppreps que estava sendo desenvolvido no Brasil.

Quando publicou esse trabalho, Ferreira já tinha sido promovido a diretor do Departamento de Desenvolvimento de Recursos Humanos da Opas, momento em que suas atividades – antes dedicadas somente à educação médica e de saúde pública – passaram a incluir outras profissões da saúde. Nesse posto, que ocupou por 21 anos, escreveu diversos trabalhos sobre a educação médica e os modelos de organização administrativa das instituições de ensino, sobre experiências que conheceu – como a estratégia chinesa dos médicos descalços –, e as que ajudou a implementar – como a organização da atenção primária em Cuba.

As reflexões sobre os modelos de cooperação continuaram na Opas. Em 1985, a organização havia criado um Programa de Residência em Saúde Internacional,^4^ que recebia anualmente em Washington dez profissionais que atuavam como assessores *juniors* em áreas selecionadas de acordo com sua atividade ou interesse profissional. O pré-requisito era que tivessem concluído um mestrado em saúde pública e, portanto, que tivessem capacidade de reflexão. Ao final dos anos 1980, os alunos e egressos desse programa propuseram a realização do seminário “A saúde internacional: um campo de estudos e prática profissional”, que discutiu criticamente o conceito de saúde internacional.

Desde os anos 1980, o pensamento liberal ortodoxo dominava as relações internacionais sob a liderança dos EUA, da Inglaterra e das instituições de Bretton Woods, e o movimento de cooperação entre os países em desenvolvimento não havia florescido muito. No fim da década, o muro de Berlim havia sido derrubado, simbolizando a derrota do socialismo e abrindo portas para a desintegração da URSS, que aconteceria ao longo de 1992. Iniciava-se a era da globalização sob a hegemonia do pensamento neoliberal. O desenvolvimento dos meios de comunicação, o surgimento da aids e de outras novas doenças, além da reemergência de antigas sob a capa de novos comportamentos, chamavam atenção para o acirramento da interdependência entre os países – e esse contexto se refletia também nas visões desse grupo de sanitaristas.

Foi nesse contexto internacional que ocorreu em Quebec, Canadá, em março de 1991, o evento proposto pelos alunos da Residência em Saúde Internacional da Opas, reunindo cerca de quarenta pessoas entre latinos, estado-unidenses e canadenses. A tônica do encontro foi a tentativa de superar o enfoque clássico da saúde internacional, que teria sido construído a partir da intenção de capacitar o outro, o diferente, partindo-se da ideia de um Norte, implícito, que deteria o saber. Nessa aproximação, os países em desenvolvimento (ou minorias sociais de países desenvolvidos) apareceriam como um sistema total, ignorando-se as relações, especificidades e subjetividades que se desenvolviam no interior desses sistemas e que de certa forma o determinavam como tal. As práticas realizadas sob esse enfoque seriam, portanto, respostas homogêneas e descontextualizadas impostas a realidades heterogêneas, além de assistencialistas, o que teria impedido o desenvolvimento ou o fortalecimento da capacidade científica e tecnológica no interior dos países subdesenvolvidos.

Para superar esse enfoque, os participantes do seminário afirmaram que o objetivo primordial de uma nova abordagem da cooperação internacional em saúde seria o fortalecimento da *autocapacidad* dos países, substituindo-se a ideia de dependência pela de interdependência, o que impulsionaria medidas bilaterais e multilaterais em substituição ao modelo de centro-periferia. Ferreira ficou encarregado de escrever o relatório final do encontro, junto com Charles Godue, médico canadense do Departamento de Saúde Comunitária do Hospital Maisonneuve-Rosemont de Montreal; Laura Nervi, antropóloga argentina consultora do Programa de Recursos Humanos da Opas; e Maria Isabel Rodrigues, médica salvadorenha, coordenadora do Programa de Residência da Opas.

Um dos participantes do seminário foi Paulo Buss, que, na época, era diretor da Escola Nacional de Saúde Pública da Fiocruz e, em sua fala no evento, caracterizou o mundo que se transformava naquele início da última década do século XX como um momento em que se ampliavam as desigualdades entre nações e no interior delas. Ressaltou as consequências da crise da dívida para os países em desenvolvimento, as milhões de pessoas em situação de pobreza, a injustiça na divisão internacional da produção, que se acirrava com a transnacionalização das empresas. Argumentou que o conceito de saúde internacional era determinado pelas concepções sobre o processo saúde-doença, sobre como se deviam organizar as sociedades e as relações internacionais; que o “reducionismo biológico” servia como base para a ideia de que se deveria combater “enfermidades tropicais” em países subdesenvolvidos, reproduzindo uma ideia de “trópicos enfermos” – característica de um pensamento de fins do século XIX. Comentou ainda que, pela visão de agências internacionais e principais doadores, os países que necessitavam de ajuda formavam um conjunto único, sem histórias, culturas e conjunturas próprias. Para ele, os programas de saúde internacional deveriam incorporar conceitos, marcos teóricos e metodológicos elaborados pela medicina social latino-americana, baseadas em utopia e solidariedade (Buss, 1992).

Buss e Ferreira haviam se conhecido em 1979, em uma reunião em Brasília, quando se discutia a criação da Associação Brasileira de Saúde Coletiva (Abrasco). No ano seguinte à reunião de Quebec, em 1992, Ferreira convidou Paulo Buss para integrar uma missão da Opas à URSS, a convite de autoridades daquele país, para conhecer iniciativas de formação de pessoal paramédico. Quando Ferreira decidiu se aposentar da Opas, aos 62 anos, Paulo Buss, então vice-presidente da Fiocruz, o convidou para assumir o cargo de coordenador de Cooperação Internacional da fundação. Era 1996 quando Ferreira retornou ao Brasil.

## O trabalho na Fiocruz e a parceria com a Opas no marco da política externa brasileira

Ao regressar, Ferreira passou alguns meses na Assessoria de Cooperação Internacional da Fiocruz, uma área que se dedicava principalmente à organização burocrática de processos de afastamento do país de pesquisadores que realizavam intercâmbios e outras atividades no exterior. Passados alguns meses, Paulo Buss deixou o cargo de vice-presidente da Fiocruz e voltou às atividades na Ensp. Ferreira o acompanhou e iniciou atividades na escola que havia ajudado a conceber trinta anos antes. Em 1998, Buss foi eleito diretor da Ensp e Ferreira passou a ser seu braço direito para assuntos internacionais. Na presidência da Fiocruz e na Ensp, as atividades de Ferreira eram voltadas para o fomento dos intercâmbios acadêmicos, o estabelecimento de convênios com instituições de renome internacional, como o National Institute of Health dos EUA e o governo canadense, entre outros, que foram responsáveis pela introdução de novos campos de conhecimento na escola e na fundação (Kastrup, 2015).

Em 2000, Paulo Buss foi eleito presidente da Fiocruz, e a Assessoria Internacional da fundação ficou a cargo de Ferreira. Buss tinha especial interesse em que a Fiocruz ocupasse uma posição relevante na saúde global e encontrou um contexto favorável, quando a discussão em torno dos Objetivos de Desenvolvimento do Milênio (ODM) e a entrada dos países emergentes, entre eles o Brasil, na disputa por poder nas relações internacionais impulsionavam uma retomada da cooperação entre países em desenvolvimento (Cabana, 2014).

Os ODM tinham a cooperação internacional como principal meio de consecução e era necessário que a prática funcionasse bem. Contudo, havia muitas críticas aos modelos adotados desde o fim da Segunda Guerra, como, por exemplo, que a cooperação havia sido muito ideologizada e dirigida por interesses dos países envolvidos na Guerra Fria. Muitos analistas argumentavam que os projetos, em geral, eram ineficazes, pois se baseavam em soluções desenvolvidas em realidades diferentes das quais eram implementados, ou que a cadeia da ajuda tinha virado um fim em si e negligenciava as opções políticas dos países receptores, ou, ainda, que as exigências e os diferentes modelos de prestação de contas adotados pelos países doadores representavam uma burocracia enorme para as estruturas administrativas dos países que necessitavam da ajuda (Foster, 2000; Sogge, 2004). Essas críticas fortaleciam o discurso da CSS, que se pretendia um modelo mais eficaz. Nesse contexto, os países desenvolvidos passaram a promover debates sobre como melhorar a prática da cooperação internacional.

Ao longo da primeira década do século XXI, a Organização para a Cooperação e o Desenvolvimento, que reunia esses principais países, promoveu uma série de reuniões – em Roma (2003), Paris (2005), Acra (2008) e Busan (2011) – que buscaram soluções para o problema da ineficácia da cooperação e culminaram com recomendações: as ações deveriam ser coordenadas no território dos receptores da ajuda; os doadores deveriam promover ações que fossem alinhadas às políticas nacionais dos países receptores; as diferentes ações promovidas por diferentes doadores deveriam ser harmonizadas entre si, evitando-se fragmentação.

Nesse ambiente de debates sobre a prática da ajuda internacional, os países emergentes encontraram espaço para retomar algumas perspectivas do MNA que haviam perdido força nas décadas anteriores, e passaram a criticar a cooperação promovida por países do Norte e a afirmar que suas ações eram mais eficazes, pois conheciam os desafios dos países mais pobres, dado que, apesar de serem economias em crescimento, ainda conviviam com desafios similares. Declaravam que sua cooperação era baseada em ajuda mútua, sem imposição de soluções ou de condicionalidades, como as compras de produtos ou a adoção de políticas sugeridas pelos países doadores. Eles buscavam se diferenciar dos países mais desenvolvidos evitando expressões como doador, receptor, assistência e ajuda, preferindo a referência à parceria e à cooperação (Esteves et al., 2011).

Foi aproveitando esse contexto que o Brasil desenvolveu as estratégias de política exterior que seriam adotadas ao longo da primeira década do século XXI. Entre 2003 e 2010, a política externa brasileira pretendeu conformar o Brasil como uma potência emergente e buscar uma posição de liderança no Sul global e, para isso, adotou uma estratégia de diversificação de parcerias, aproximando-se de outros países em desenvolvimento e enfocando a CSS como um meio de ampliar seu poder e prestígio nas relações internacionais (Vigevani, Cepaluni, 2007; Saraiva, 2007, 2013). Ao mesmo tempo, a saúde se conformava como um tema interessante para a política externa dos países: por um lado, pela ameaça que a disseminação de doenças representava à segurança dos países, e, por outro, por movimentos que buscavam conformar a saúde como objeto da solidariedade humana e justiça social (Fidler, 2007).

Internamente, no campo setorial, a chegada de Lula à presidência em 2003 representou um momento de otimismo para os sanitaristas progressistas que haviam participado, em diferentes momentos e de diversas formas, do movimento da medicina social latino-americana e do movimento pela reforma sanitária brasileira. A ascensão de um governo de orientação progressista significou que sanitaristas oriundos desses movimentos passaram a ocupar cargos no Ministério da Saúde, como, por exemplo, José Saraiva Felipe, um médico mineiro que havia participado de um importante projeto para a reforma sanitária em Montes Claros, Minas Gerais, que assumiu o Ministério da Saúde do Brasil em 2005. Felipe convidou o também médico mineiro Francisco Eduardo Campos, que havia sido assessor de Ferreira na Opas entre os anos de 1989 e 1996, para liderar a Secretaria de Gestão do Trabalho e da Educação em Saúde do ministério (SGTES). Nessa secretaria, um dos principais projetos de Campos foi a reorientação do ensino das profissões de saúde para a ênfase na Atenção Primária em Saúde, para o qual convidou Ferreira para participar como assessor do Ministério da Saúde.^5^ Assim, nove anos após a chegada no Brasil, Ferreira passou a associar a atividade na Assessoria de Cooperação Internacional da Fiocruz à discussão sobre o ensino das profissões de saúde, duas funções às quais havia se dedicado durante toda a sua carreira.

Com Campos na liderança da SGTES foi articulado um projeto de cooperação com a Opas, o TC-41, que viria a ser um dos pilares da política externa brasileira para a cooperação em saúde entre 2005 e 2015. Naquele momento, dada a dimensão de sua atividade institucional, a Fiocruz passou a ser a principal instituição assessora e executora da cooperação em saúde no marco da política externa brasileira, e esse programa ficou sob a coordenação de José Roberto Ferreira. A parceria com Paulo Buss, que desde 2000 ocupava o cargo de presidente da Fiocruz, foi intensa. Buss dedicava-se principalmente à diplomacia da saúde, participando de diversos *boards* da OMS, da Opas, da Comunidade dos Países de Língua Portuguesa e, posteriormente, da União de Nações Sul-Americanas; e Ferreira era encarregado de colocar a cooperação em prática, discutindo com lideranças dos países parceiros e técnicos do Ministério da Saúde e da Agência Brasileira de Cooperação do Ministério das Relações Exteriores do Brasil o escopo dos projetos, reunindo e orientando técnicos e pesquisadores da Fiocruz que executavam atividades em solo estrangeiro.

A experiência em torno desse movimento foi sistematizada no artigo que apresentou a ideia da CSSE, cuja autora principal, Celia Maria de Almeida, era naquele momento diretora do escritório da Fiocruz em Maputo, Moçambique. Celia é médica, pesquisadora aposentada da Ensp e havia dedicado parte da sua vida profissional ao trabalho em Moçambique, com atividade de pesquisa voltada ao estudo das reformas do setor da saúde em diferentes países e de sistemas de saúde em perspectiva comparada.

O artigo que apresenta a ideia da CSSE, que tem Almeida e Buss entre os autores (Almeida et al., 2010), ilustra o entrelaçamento entre o pensamento sobre o sanitarismo internacional, cultivado na Fiocruz pelo menos desde os anos 1990, e a política governamental de tomar a cooperação em saúde como elemento de política externa. O texto não representa uma tentativa de teorização *a priori*, mas apresenta um processo em curso organizado sobre as bases teóricas que os autores sistematizam no texto. O trabalho parte de uma revisão histórica crítica da cooperação internacional e da cooperação internacional em saúde, que busca explicitar como as ações da saúde internacional estiveram condicionadas por interesses e conjunturas que não contribuíram para que a ajuda chegasse a promover a melhora das condições de saúde das populações que dela necessitavam. Os autores citam como determinantes dessa realidade a hegemonia neoliberal que se consolidou no mundo a partir da década de 1980, com as políticas de ajuste estrutural prescritas pelo Banco Mundial, a epidemia de aids que sobrecarregou os sistemas de saúde, as migrações de profissionais de saúde de países mais pobres para os países do Norte, além do modelo “tradicional” de ajuda Norte-Sul em saúde orientado pelo combate às enfermidades específicas.

A partir desses argumentos, defendem uma maneira de pensar a cooperação em saúde baseada nos preceitos da CSS com o objetivo central de promover a estruturação dos sistemas de saúde. O modelo seria centrado no desenvolvimento de capacidades internas dos países e focado nas instituições consideradas estruturantes para os sistemas de saúde como Ministérios da Saúde, Escolas de Saúde Pública, Institutos Nacionais de Saúde e Escolas Técnicas. Para os autores, essa concepção estava baseada na construção de capacidades para o desenvolvimento, o que significaria uma inovação, por integrar formação de recursos humanos e desenvolvimento institucional e por propor que se buscasse explorar capacidades e recursos do próprio país (Almeida et al., 2010, p.28).

Em suma, a CSSE integrava concepções sobre como fazer cooperação, baseadas nos ideais da CSS, ao que se deveria fazer em termos de cooperação na área da saúde. Isto é, juntava a proposta de construir parcerias, intercambiar experiências, compartilhar resultados e aprendizagem, ao entendimento de que se deveria desenvolver capacidades a partir da mobilização do conhecimento local, baseado nas realidades próprias, de modo que os países receptores desenvolvessem agendas autônomas para o seu desenvolvimento sanitário.

A crítica aos modelos da saúde internacional está presente mais explicitamente em outro artigo assinado por Buss e Ferreira, publicado no mesmo número da revista que incluiu o texto sobre a cooperação estruturante. No texto, os autores (Buss, Ferreira, 2010a) descreveram o modelo dominante da cooperação internacional, apontando que, neste, os países desenvolvidos imporiam suas próprias visões de mundo aos menos desenvolvidos. Também destacaram, como no artigo de Ferreira de 1976, a diferença entre assistência e cooperação, e, incorporando os princípios definidos nos debates sobre a eficácia da ajuda internacional, desaprovaram a descoordenação e a sobreposição de projetos em países pobres. Eles atualizaram a crítica às agendas verticais, argumentando que os principais atores da saúde global adotavam uma comunicação unidirecional, ignorando processos já implementados nos países nos quais os projetos eram desenvolvidos.

Os programas verticais não contribuem para o fortalecimento do sistema como um todo; ao contrário, levam à fragmentação e à debilidade do mesmo, seja pelo recrutamento do melhor pessoal disponível no país, seja por se concentrarem em certas áreas, abandonando outras áreas prioritárias (Buss, Ferreira, 2010a, p.96).

Os autores retomam, assim, na saúde global, um dilema eivado de concepções ideológicas que permeavam a saúde internacional: o combate verticalizado às doenças *versus* a prevenção e a estruturação da atenção à saúde (Cueto, 2004), e acusam as parcerias público-privadas na saúde de serem provedoras de assistência tradicional, entendida como ineficaz. Tal perspectiva revela a influência de uma perspectiva crítica presente no movimento da medicina social latino-americana.

Nos primeiros anos da segunda década de 2000, como coordenador adjunto do Centro de Relações Internacionais em Saúde da Fundação Oswaldo Cruz, Ferreira publicou diversos artigos sobre as experiências de CSS em saúde, desenvolvidas pela fundação, principalmente junto à União de Nações Sul-Americanas e à Comunidade dos Países de Língua Portuguesa (Buss, Ferreira, 2010b, 2011, 2012, 2017; Buss, Ferreira, Hoirisch, 2011; Ferreira et al., 2016; Ferreira, Fonseca, 2017).

Esse conjunto de trabalhos caracterizou a última etapa da trajetória profissional de Ferreira, marcada pela defesa do conceito de cooperação Sul-Sul estruturante e pela busca por demonstrar sua aplicação e seu desenvolvimento nos espaços privilegiados pelo Brasil na CSS.

Se a atuação brasileira na cooperação internacional em saúde a partir de 2005 ainda carece de maiores investigações que confrontem discurso e prática (Russo, Cabral, Ferrinho, 2013), a última etapa da trajetória profissional de Ferreira é marcada pelo esforço por propagar os marcos conceituais que a orientaram, desenvolvidos ao longo de sua trajetória, e que encontraram espaço político para a concretização por meio de projetos e alianças regionais no marco da política externa brasileira. Ainda que a estratégia tenha sido gradativamente descontinuada, ao longo da segunda década dos anos 2000, o conjunto de projetos desenvolvidos e a construção, na Fiocruz, de um grupo de profissionais dedicados a trabalhar na cooperação técnica internacional e, ao mesmo tempo, a desenvolver o conceito por meio de debates e publicações acadêmicas tornaram possível a disseminação do conceito de cooperação Sul-Sul estruturante na própria instituição e em outras instâncias do Ministério da Saúde do Brasil e da diplomacia da saúde brasileira (Kastrup, 2023).

## Considerações finais

Apresentamos o papel da trajetória de José Roberto Ferreira para a construção do conceito de cooperação Sul-Sul estruturante, que se consolidou como orientador para as atividades de cooperação técnica internacional em saúde do Brasil, durante a primeira década dos anos 2000. O início dessa trajetória foi marcado pela preocupação com a construção de escolas de medicina que incorporassem a realidade dos serviços básicos de saúde como parte da formação do profissional médico, o que levou Ferreira a uma posição na Opas, desde onde, encarregado da cooperação regional e inspirado pelo movimento de aproximação entre países em desenvolvimento, construiu uma concepção de cooperação técnica em saúde que tinha como finalidade a superação da dependência dos países receptores das ações. Tal perspectiva partia da proposta de se basear na realidade local e no saber autóctone para a construção de soluções para a saúde. Sob essa perspectiva, o papel do agente internacional, ou do país que ofertava sua suposta *expertise*, seria de escuta ativa e de fortalecimento dos quadros e das autoridades locais.

Tais ideias de um modelo de cooperação emancipador, gestado na Divisão de Recursos Humanos da Opas em Washington, guarda estreita relação com o movimento da medicina social latino-americana, também impulsionada desde ali e que propunha a formação de médicos e sanitaristas sensíveis às realidades locais e sociais das populações. Daí a construção de uma concepção de saúde internacional também de caráter emancipatório.

Segundo a nossa perspectiva, tais ideias foram transpostas à saúde global no bojo de um governo progressista no Brasil, que valorizou a CSS e a saúde como instrumentos da política externa. A posição de Ferreira na Fiocruz e o papel que a fundação assumiu no desenvolvimento dessa política externa possibilitaram tal transposição e a formulação do conceito de cooperação Sul-Sul estruturante, que teve papel fundamental também nas ideias e experiências dos outros autores do artigo. A proposta de se fortalecer instituições e formar recursos humanos como um meio de promover a autonomia e a sustentabilidade dos sistemas de saúde de países receptores apontam para o papel central de José Roberto Ferreira.

## Data Availability

Não estão em repositório.

## NOTAS

1 Assinam o trabalho Celia de Almeida, que, em 2010, era chefe do escritório da Fiocruz na África; Paulo Buss, chefe do Centro de Relações Internacionais em Saúde da Fiocruz (Cris); Luís Eduardo Fonseca, médico e técnico do Cris; Rodrigo Pires de Campos, do Instituto de Relações Internacionais da Universidade de Brasília; e José Roberto Ferreira, então diretor-adjunto do Cris.

2 Durante a primeira fase de sua carreira, Ferreira publicou 18 trabalhos científicos sobre cirurgia entre artigos, publicações e conferências.

3 Em 1955 e 1956, a Opas realizou dois seminários históricos em Viña del Mar, Chile, e em Tehuacán, México, que são considerados marcos da introdução do tema da medicina preventiva na América Latina.

4 Esse programa se desenvolve até o presente com o nome de “Programa de Líderes em Saúde Internacional”.

5 O projeto que Ferreira passou a integrar foi o Pró-Saúde.
